# Study of depression and its associated factors among women living with HIV AIDS in coastal South India

**DOI:** 10.1186/1742-4690-9-S1-P137

**Published:** 2012-05-25

**Authors:** B Unnikrishnan, V Jagannath, JT Ramapuram, S Hegde

**Affiliations:** 1 Kasturba Medical College (Manipal University), Mangalore, India

## Backgound

Depression is one of the most prevalent psychiatric diagnoses seen in HIV-positive individuals. Women with HIV are about seven times more likely to be depressed than those who are not infected, Depressive symptoms in women are associated with impaired adherence to antiretroviral therapy (ART), higher HIV plasma viral loads, higher mortality, less social support and a worse quality of life.The study was carried out with the objective to assess the sociodemographic and clinical correlates of Depression among Women living with HIV/AIDS.

## Method

The crossectional study was carried out in one Public and one Private hospital in Mangalore, Coastal South India. The study subjects included 137 HIV Positive women enrolled after obtaining written informed consent. The data was collected using a pretested semi structured proforma which elicited information about socio demographic variables, HIV related clinical and laboratory data .Depression was assessed using BDI (Becks Depression Inventory), Lubben Social Network Scale was used to assess social support. The data was analyzed using SPSS Version 11.5, for univariate analysis chisquare test and t test were used and for multivariate analysis step wise logistic regression model was used. The study was approved by the Institutional Ethics Committee of Kasturba Medical College, Mangalore.

## Results

Among 137 HIV positive women, 51% were depressed. The mean (SD) age of the subjects was 35 (7) years. The mean (SD) CD4 count among those with Depression was 328 (125). Majority (63%) of the subjects were not on ART and 16% were having moderate to high risk for isolation. In multivariate regression model, living in rural area, Widowed, Lower socio economic status were significantly associated with depression.

## Conclusion

Depression is highly prevalent among women living with HIV and there is a need to incorporate mental health services as an integral component of HIV care.

